# Lorlatinib overcomes alectinib‐induced hemolytic anemia in an ALK fusion positive non‐small‐cell lung cancer patient with severe tumor‐associated liver failure: A case report

**DOI:** 10.1111/1759-7714.15487

**Published:** 2024-11-11

**Authors:** Kei Kunimasa, Akito Miyazaki, Motohiro Tamiya, Takako Inoue, Takahisa Kawamura, Tsunehiro Tanaka, Shun Futamura, Kiyohide Komuta, Shigenori Nagata, Keiichiro Honma, Kazuyoshi Ohkawa, Kazumi Nishino

**Affiliations:** ^1^ Department of Thoracic Oncology Osaka International Cancer Institute Osaka Japan; ^2^ Department of Diagnostic Pathology & Cytology Osaka International Cancer Institute Osaka Japan; ^3^ Department of Hepatobiliary and Pancreatic Oncology Osaka International Cancer Institute Osaka Japan

**Keywords:** ALK fusion, alectinib, hemolytic anemia, liver failure, lorlatinib

## Abstract

Hemolytic anemia is a rare and unique complication of alectinib, not observed with other anaplastic lymphoma kinase (ALK) inhibitors. Here, we present a case of an ALK fusion‐positive non‐small‐cell lung cancer (NSCLC) patient who developed liver failure due to diffuse liver metastasis at initial diagnosis. Treatment was initiated with low‐dose alectinib, but the patient developed severe hemolytic anemia. Switching to lorlatinib allowed for the continuation of ALK inhibitor therapy and successful tumor reduction. ALK inhibitors are crucial for ALK fusion‐positive NSCLC patients. Managing severe side effects by switching medications is essential to maintain effective therapy. In this case, lorlatinib effectively controlled the tumor and improved the patient's liver function and performance status. This case highlights the importance of adapting treatment strategies to manage adverse effects while ensuring the continued use of ALK inhibitors for optimal patient outcomes.

## Graphical abstract

Alectinib can cause hemolytic anemia, a rare side effect. We report a case of an anaplastic lymphoma kinase (ALK) fusion‐positive non‐small‐cell lung cancer patient who developed severe hemolytic anemia after starting low‐dose alectinib due to liver failure from diffuse liver metastasis. Switching to lorlatinib allowed continuation of ALK inhibitor therapy, resulting in tumor reduction and improved liver function. This case underscores the importance of managing side effects by switching medications to maintain effective treatment.

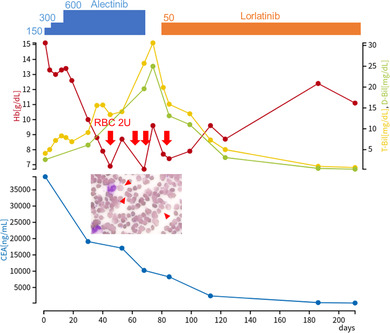

## INTRODUCTION

Lorlatinib, a third‐generation anaplastic lymphoma kinase (ALK) inhibitor with a macrocyclic structure, is designed to enhance central nervous system (CNS) penetration. In a phase III trial comparing lorlatinib with crizotinib for ALK‐positive non‐small‐cell lung cancer (NSCLC) patients, lorlatinib demonstrated a significant extension in progression‐free survival (PFS).^1^ Recently published 3‐year follow‐up data reported sustained PFS benefits and effective control of brain metastases.^2^ Characteristic toxicities of lorlatinib include hyperlipidemia and CNS adverse events.^2^

Here, we report a case of an ALK‐positive NSCLC patient with significant liver metastases and tumor‐induced liver failure at initial presentation. Although treatment with alectinib was initiated, the patient developed alectinib‐induced hemolytic anemia. Switching to lorlatinib resulted in successful tumor suppression and improvement of liver failure.

## CASE PRESENTATION

**FIGURE 1:**
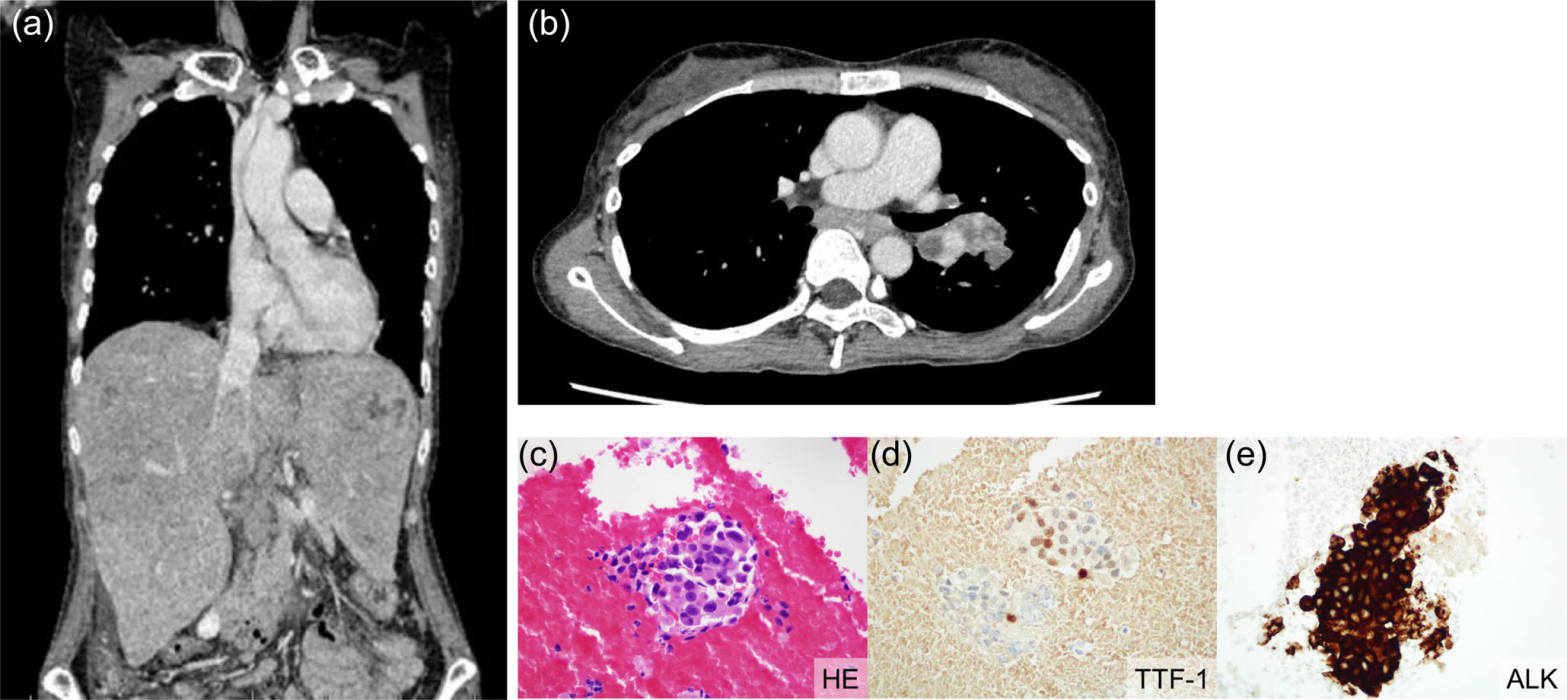
Coronal section of the contrast‐enhanced chest and abdominal CT scan showing a significantly enlarged liver (a). Transverse section of the contrast‐enhanced chest CT scan demonstrating enlarged mediastinal lymph nodes (b). Hematoxylin and eosin staining of a mediastinal lymph node biopsy obtained by endobronchial ultrasound‐guided transbronchial needle aspiration (EBUS‐TBNA) (c). Clusters of cells with atypical nuclei among red blood cells are observed, and these nuclei are positive for TTF‐1 (d). ALK staining (D5F3, Ventana Medical Systems, Inc., Tucson, AZ) of the same cells showing strong positivity (e). CT, computed tomography.

A 50‐year‐old man, who was a never smoker, presented to our hospital with complaints of jaundice and abdominal distension. She had no significant past medical history and was not on any medications. Contrast‐enhanced chest and abdominal computed tomography (CT) scans revealed diffuse hepatomegaly (Figure 1a). While no apparent tumorous lesions were observed in the lung fields, mediastinal lymphadenopathy was noted (Figure 1b). Serum biochemistry showed elevated hepatobiliary enzymes with T‐Bil 4.1 mg/dL, Alb 2.5 g/dL, AST 356 U/L, ALT 526 U/L, ALP 1110 U/L, PT‐INR 1.9, and LDH 566 U/L. Based on these data and the absence of hepatic encephalopathy and ascites, the patient was classified as Grade C according to the Child‐Pugh classification. Tumor marker tests revealed markedly elevated levels of carcinoembryonic antigen (CEA) 39 019.9 ng/mL, NSE 296.5 ng/mL, and CA19‐9 > 100 000 U/mL. An endobronchial ultrasound–guided transbronchial needle aspiration (EBUS‐TBNA) of the mediastinal lymph nodes revealed malignant cells positive for TTF‐1 (Figure 1c,d) and strong positivity for ALK (Figure 1e), and the Amoy lung cancer panel detected an ALK fusion, confirming a diagnosis of ALK‐positive lung adenocarcinoma with clinical stage IVB.

**FIGURE 2:**
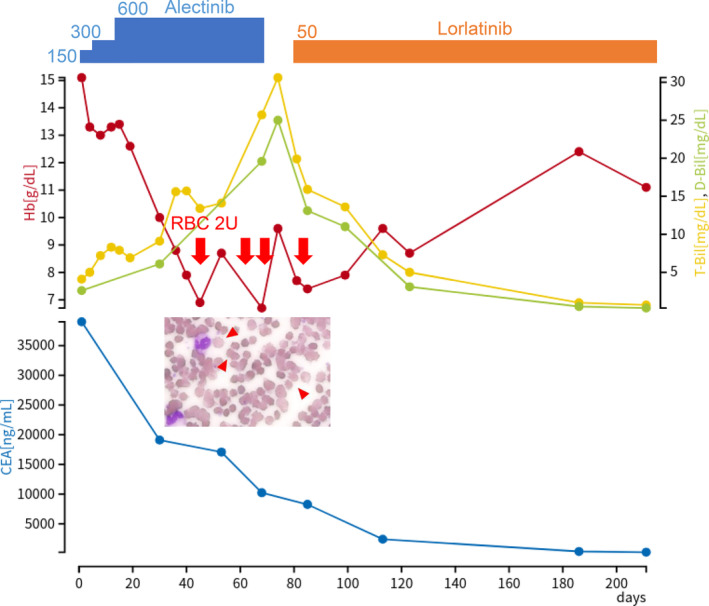
The course after initiating alectinib treatment. The left vertical axis represents hemoglobin (Hb, g/dL) and CEA (ng/mL) levels, while the right vertical axis shows T‐Bil (mg/dL) levels. Red arrows indicate red blood cell (RBC) transfusions, with 2 units transfused per session. The peripheral blood smear image corresponds to the period of anemia, with red arrowheads indicating fragmented red blood cells. CEA, carcinoembryonic antigen.

At the initial presentation, the patient had a performance status (PS) of 3 and liver failure due to diffuse liver metastasis. Treatment was initiated with alectinib at the minimum dose of 150 mg, with a gradual dose escalation planned. The dose was increased to 300 mg and then 600 mg over 5‐day intervals. However, there was no improvement in the abdominal distension. While serum CEA levels decreased, T‐Bil levels continued to rise, reaching 24.1 mg/dL, and indirect bilirubin was 5.3 mg/dL. Concurrently, the patient developed worsening anemia, requiring multiple red blood cell transfusions. Peripheral blood smears revealed fragmented red blood cells, reticulocyte was 19.1‰, Coombs test was negative, haptoglobin was less than 10 mg/dL, and ADAMTS13 activity was greater than 10%, leading to a diagnosis of alectinib‐induced hemolytic anemia (Figure 2).

**FIGURE 3:**
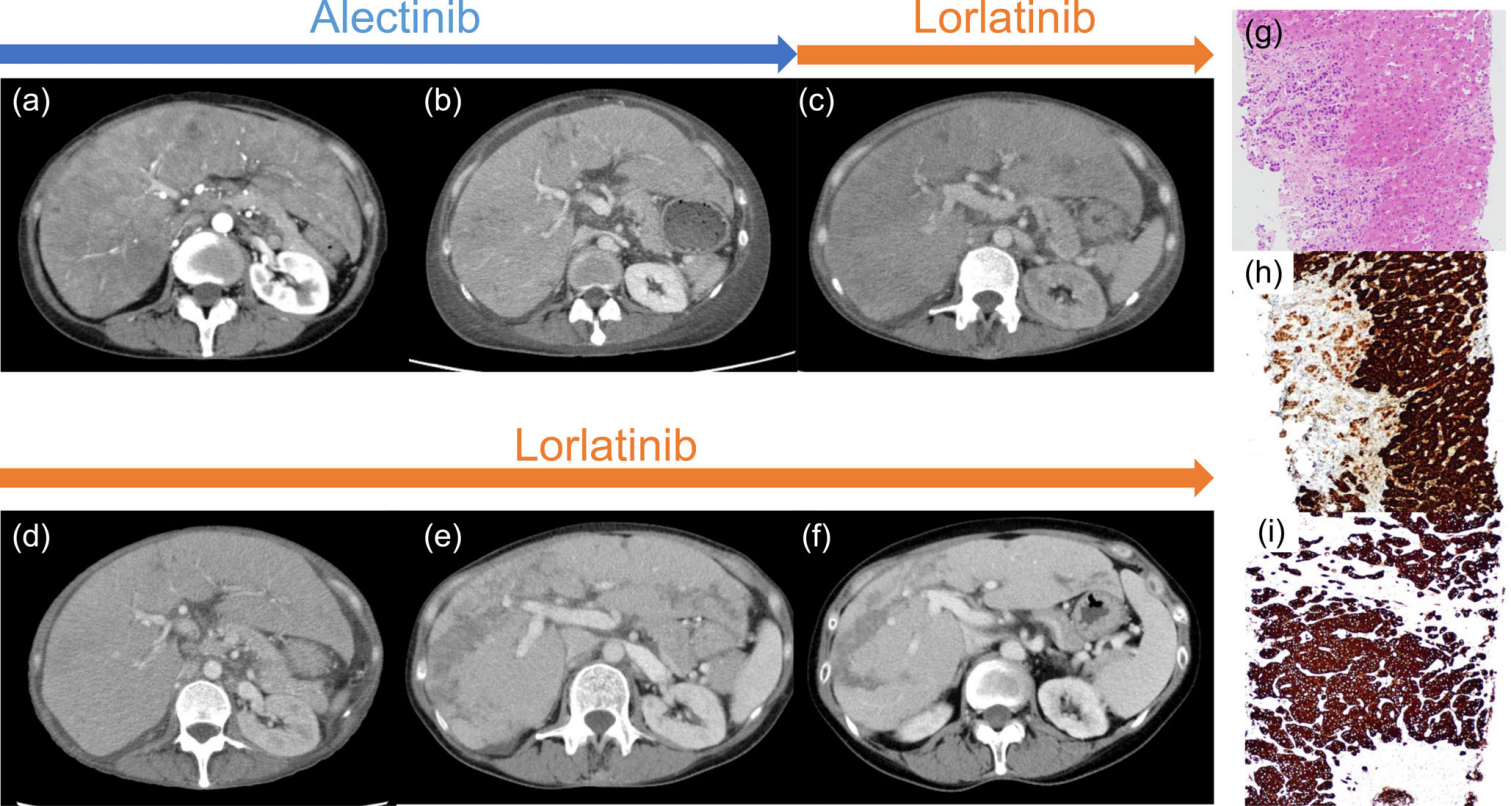
Time course of transverse sections of the liver on contrast‐enhanced CT after initiating ALK inhibitors. (a) Transverse section of the liver on contrast‐enhanced CT at initial presentation. (b) Approximately 1 month after initiating alectinib. (c) At the time of lorlatinib initiation after discontinuing alectinib. (d) One month after starting lorlatinib. (e) Two months after starting lorlatinib. (f) Four months after starting lorlatinib. Pathological analysis of liver needle biopsy specimens with hematoxylin and eosin staining (g) and TTF‐1 staining (h), showing scattered cells with TTF‐1 positive atypical nuclei against a background of hepatocytes. Strong staining confirmed in tumor cells by ALK staining (i). ALK, anaplastic lymphoma kinase; CT, computed tomography.

Upon switching from alectinib to oral lorlatinib 50 mg, the patient experienced an improvement in anemia and a decrease in T‐Bil levels (Figure 2). Over approximately 6 months, imaging revealed a reduction in hepatomegaly and an improvement in intrahepatic necrosis (Figure 3a–f). The Child–Pugh classification also showed improvement from class C to A, and PS became 0. A liver biopsy was performed once the liver failure had improved, and pathological examination confirmed liver metastasis of the lung cancer (Figure 3g–i). When the lorlatinib dose was increased to 75 mg, the patient developed grade 2 peripheral neuropathy, leading to the decision to maintain a 50‐mg dose. The patient is currently able to continue the treatment with lorlatinib.

## DISCUSSION

ALK inhibitors are extremely valuable drugs for ALK‐positive NSCLC patients, significantly contributing to prolonged prognosis.^2^ Among lung cancer drugs, their impact on extending survival is arguably the greatest. In this case, despite the poor prognosis due to tumor‐induced liver failure and poor PS, low‐dose alectinib was chosen. However, the patient developed hemolytic anemia, necessitating a switch in medication. While lorlatinib is known for its higher toxicity, it was safely introduced at 50 mg in this case, successfully controlling the tumor. The patient's liver failure improved, and PS improved, allowing the patient to live a normal daily life comparable to that of a healthy individual.

Alectinib, compared to brigatinib and lorlatinib, is known for having the least toxicity while still offering substantial antitumor efficacy.^3^ Alectinib and lorlatinib have distinct toxicity profiles.^1^, ^4^, ^5^ Alectinib is commonly associated with mild side effects such as fatigue, constipation, peripheral edema, and myalgia, whereas lorlatinib can cause more significant CNS‐related adverse events, including mood changes, cognitive impairment, and peripheral neuropathy. Additionally, lorlatinib is known to cause hyperlipidemia, which requires careful monitoring. Despite these differences, both agents share some common toxicities, including elevated liver enzymes and interstitial lung disease, though the incidence and severity can vary between the two. In the ALEX trial,^5^ which evaluated alectinib, hepatotoxicity was observed but was generally mild to moderate in severity. The incidence of increased liver enzymes (AST/ALT) of any grade was approximately 15% for AST and 14% for ALT, with grade 3–4 elevations occurring in 5% for AST and 6% for ALT.^5^ Most cases were manageable with dose modification or interruption. In the CROWN trial,^1^ which investigated lorlatinib, the frequency of hepatotoxicity was relatively higher. The incidence of increased liver enzymes was reported at around 20% for AST and 23% for ALT for all grades, with grade 3–4 elevations occurring in 8% for both AST and ALT. This indicates that while both agents can cause hepatotoxicity, lorlatinib has a slightly higher incidence and severity of liver enzyme elevations compared to alectinib. Therefore, more vigilant monitoring of liver function may be necessary when using lorlatinib.

However, a unique toxicity observed with alectinib is hemolytic anemia.^6^ Alectinib is metabolized via CYP3A4, and it is known that in cases of liver failure, the blood concentration of alectinib can increase. The standard dose in Japan is 300 mg orally twice daily.^4^ For patients with mild hepatic impairment (Child‐Pugh A), no dose adjustment is necessary. However, for those with moderate to severe hepatic impairment (Child‐Pugh B or C), the recommended dose is reduced to 200 mg orally twice daily. For lorlatinib, the dose adjustment guidance is less specific. In patients with mild to moderate hepatic impairment (Child–Pugh A or B), no specific dose adjustments are recommended, but close monitoring is essential due to the increased risk of hepatotoxicity. For patients with severe hepatic impairment (Child–Pugh C), the safety of lorlatinib has not been established, and its use should be approached with caution. In this case, despite administering alectinib at the minimum dose of 150 mg, the presence of liver failure due to tumor metastasis might have led to an abnormal increase in blood concentration. To date, there have been no reports of drug‐induced hemolytic anemia with lorlatinib, which is also metabolized by CYP3A4. Therefore, it is possible that the nature of the drug itself, rather than its blood concentration, contributes to the development of hemolytic anemia.

Diffuse metastasis from solid tumors is known to cause acute liver failure.^7^ In this case, a liver biopsy confirmed that the liver failure was due to liver metastasis from lung cancer. Once liver failure occurs, initiating any form of anticancer therapy becomes extremely challenging, and the prognosis is generally very poor. However, molecular targeted therapies for driver mutations can still be effective even in patients with poor PS, potentially improving PS and achieving rapid tumor reduction due to their high response rates. In this case, there were no alternative treatment regimens other than ALK inhibitors. The ability to continue treatment by switching ALK inhibitors was therefore of significant importance.

## CONCLUSION

We presented a case of ALK‐positive lung cancer with diffuse liver metastasis causing liver failure, where severe hemolytic anemia was induced by low‐dose alectinib. The treatment was successfully managed by switching to lorlatinib.

## AUTHOR CONTRIBUTIONS

**Kei Kunimasa:** conceptualization, investigation, methodology, writing—original draft, writing—review and editing. **Akito Miyazaki, Motohiro Tamiya, Takako Inoue, Takahisa Kawamura, Tsunehiro Tanaka, Shun Futamura, and Kiyohide Komuta:** investigation, writing—review and editing. **Shigenori Nagata and Keiichiro Honma:** investigation, methodology. **Kazuyoshi Ohkawa:** writing—review and editing. **Kazumi Nishino:** conceptualization, investigation, supervision, writing—review and editing.

## CONFLICT OF INTEREST STATEMENT

The authors declare the following financial interests/personal relationships which may be considered as potential competing interests: Dr. Kunimasa reports honoraria for lecture from AstraZeneca, Chugai Pharma, and Novartis; Dr. Tamiya reports receiving grants from Boehringer Ingelheim, Ono, MSD, Eisai, Daiichi Sankyo, Chugai, and Janssen and personal fees from Boehringer Ingelheim, Ono, MSD, Chugai, AstraZeneca, Taiho, Eli Lilly, Novartis, Asahi Kasei, Bristol‐Myers Squibb, Bayer, Amgen, Kyowa‐Kirin, and Nippon Kayaku. Dr. Ohkawa reports receiving personal fees from Chugai, AstraZeneca, Eisai, Astellas, Incyte, and Nihon Selvier. Dr. Nishino reports receiving grants from Ono, TAIHO, MSD, AbbVie, DAIICHI SANKYO, Amgen, Eisai, Sanofi, Janssen, Novartis, Pfizer, Eli Lilly, Merck, Takeda, Chugai, and Merus and personal fees from AstraZeneca, Chugai, Nippon Boehringer Ingerheim, Eli Lilly, Roche, Novartis, Pfizer, Merck, Janssen, Bristol Myers Squibb, and Nippon Kayaku. The remaining authors declare no conflict of interest.

## Data Availability

The data that support the findings of this study are available from the corresponding author upon reasonable request.
